# Fat embolism syndrome following femoral shaft fracture: A case report and diagnostic considerations

**DOI:** 10.1016/j.radcr.2024.10.126

**Published:** 2024-11-22

**Authors:** Morteza Gholipour, Mohsen Salimi, Alireza Motamedi, Fatemeh Abbasi

**Affiliations:** a Clinical Research Development Unit of Akhtar Hospital, Shahid Beheshti University of Medical Science, Tehran, Iran; b School of Medicine, Shiraz University of Medical Sciences, Shiraz, Iran; c Faculty of Medicine, Tabriz University of Medical Sciences, Tabriz, Iran; d Faculty of Medicine, Mazandaran University of Medical Sciences, Mazandaran, Iran

**Keywords:** Fat embolism syndrome, Fat embolism, Femoral fracture, Surgery, Respiratory distress, Supportive care

## Abstract

Fat embolism syndrome (FES) is a rare but serious complication that can arise after long bone fractures or orthopedic surgeries. This case report presents a 40-year-old male who developed FES following surgical fixation of a femoral shaft fracture using 2 plates. The day after surgery, the patient exhibited tachycardia, respiratory distress, and a fever of 38.5°C, initially raising concerns for pulmonary embolism. A computed tomography (CT) angiography of the lungs showed no evidence of pulmonary thromboembolism, and methylprednisolone was administered due to the suspicion of fat embolism. On the second postoperative day, petechial and purpuric lesions appeared on the neck, chest, and the surgical limb, strengthening the suspicion for FES. The patient fulfilled 2 major and 3 minor criteria for FES according to the Gurd and Wilson criteria, and scored 8 points on the Schonfeld Fat Embolism Index, indicating a high likelihood of FES. Despite these clinical signs, imaging studies did not reveal any embolic events. The patient was treated with supportive care, including oxygen therapy and anticoagulation, and his condition stabilized over the next 24 hours. He was mobilized and discharged in stable condition. This case highlights the critical need for early recognition of fat embolism syndrome (FES) in postorthopedic surgery patients, as timely diagnosis and intervention are key to preventing serious complications. Although clinical signs may not always align with imaging results, vigilant monitoring and prompt supportive care can significantly improve patient outcomes.

## Introduction

Fat embolism (FE) refers to the intravascular deposition of fat globules within the pulmonary or peripheral vasculature. Fat embolism syndrome (FES) is a rare phenomenon characterized by a set of clinical manifestations that arise following a clear triggering event and is typically marked by the classic triad of respiratory distress, neurological deficits, and petechial rash [1,2]. FES is mainly related to orthopedic trauma and often presents within 24 to 72 hours after fractures of long bones or the pelvis. It occurs in 0.5% to 2% of femoral fracture episodes [3,4]. The severity of the condition can vary, with most of the cases generally self-limiting; however, mortality rates can range from 5% to 15%, particularly in cases of acute respiratory distress syndrome (ARDS), especially if diagnosis is missed [5]. The diagnosis of FES is primarily clinical [6], although the Gurd and Wilson criteria along with the Schonfeld fat embolism index [7], are 2 widely accepted criteria for its diagnosis. Despite a few pathological processes that have been proposed, the pathophysiological mechanisms of FES are not yet fully recognized, and the condition remains poorly understood [8].

## Case presentation

**Fig. 1:**
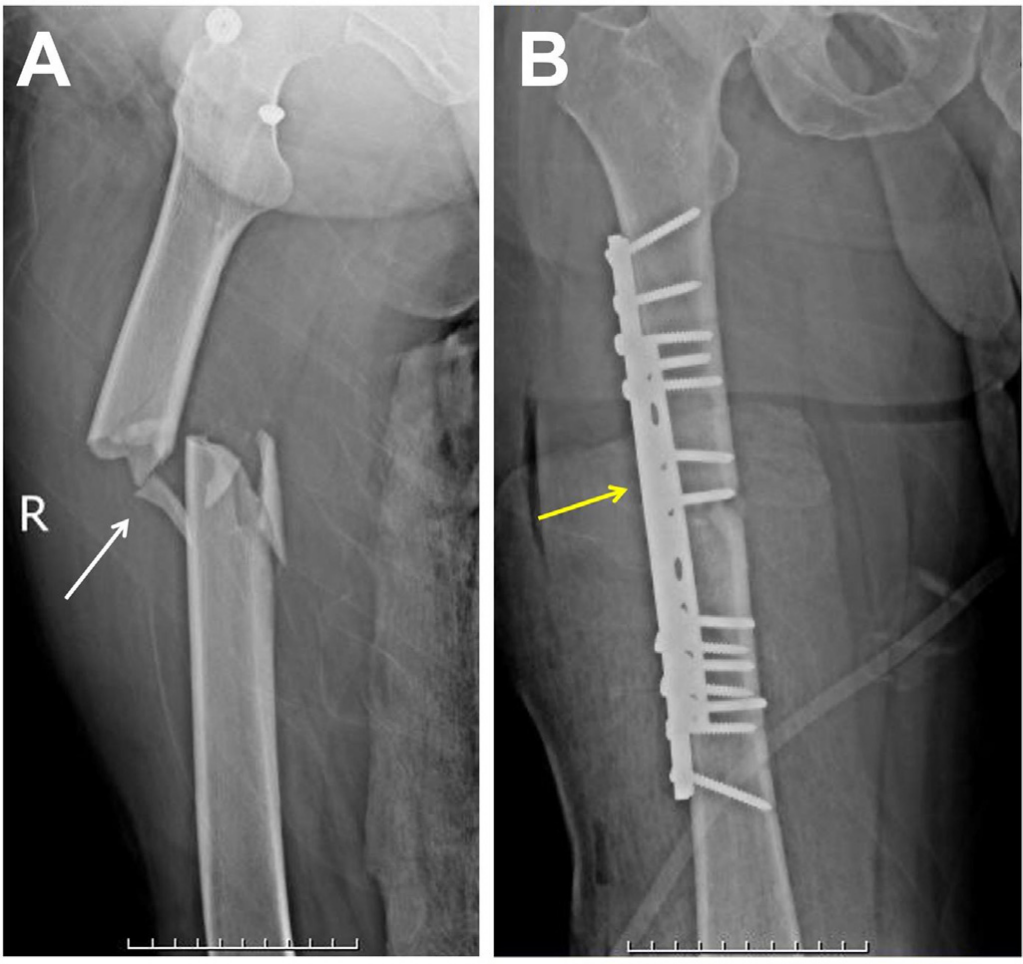
(A) Initial preoperative plain radiograph of the right femur (anteroposterior view) showing a femoral shaft fracture with medial displacement (white arrow). (B) Postoperative plain radiograph (anteroposterior view) showing open reduction and internal fixation of the right femur using 2 plates and screw fixation (yellow arrow).

A 40-year-old male with no significant medical history was brought to our trauma center after a motor vehicle accident. The patient presented with severe thigh pain, inability to bear weight, swelling, and bruising, along with noticeable deformity of the thigh, indicating a possible femoral fracture. Despite these findings, the patient was hemodynamically stable, and his vital signs were normal. A plain X-ray was taken and the diagnosis was confirmed as a femoral shaft fracture with medial displacement (Fig. 1).

The patient was taken to the operating room for open reduction and internal fixation of the right femoral shaft fracture. Under general anesthesia, the fracture site was exposed. The fracture was carefully reduced, and 2 plates was applied to the femur. Screws were inserted to secure the plates and achieve stable fixation (Fig. 1). Alignment and compression at the fracture site were confirmed. The wound was thoroughly irrigated and closed in layers. The patient tolerated the procedure well and was transferred to the recovery room in stable condition.

Twelve hours later the patient developed tachycardia (heart rate = 130) tachypnea (respiratory rate = 32) and acute respiratory distress syndrome (ARDS) along with a fever (body temperature = 38.5°C). The patient did not have altered consciousness (GCS = 15) and showed no neurological signs or confusion.

**Fig. 2:**
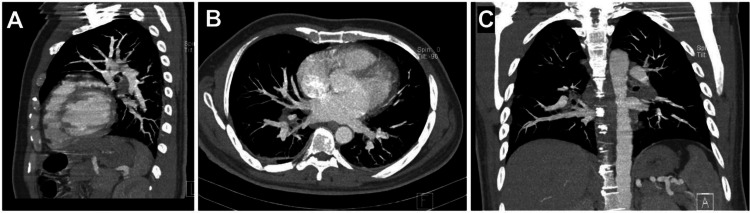
CT angiography of the lungs: (A) Sagittal view, (B) Axial view, (C) Coronal view. No evidence of pulmonary thromboembolism (PTE) or any other complications, including fat embolism syndrome (FES), was observed.

**Fig. 3:**
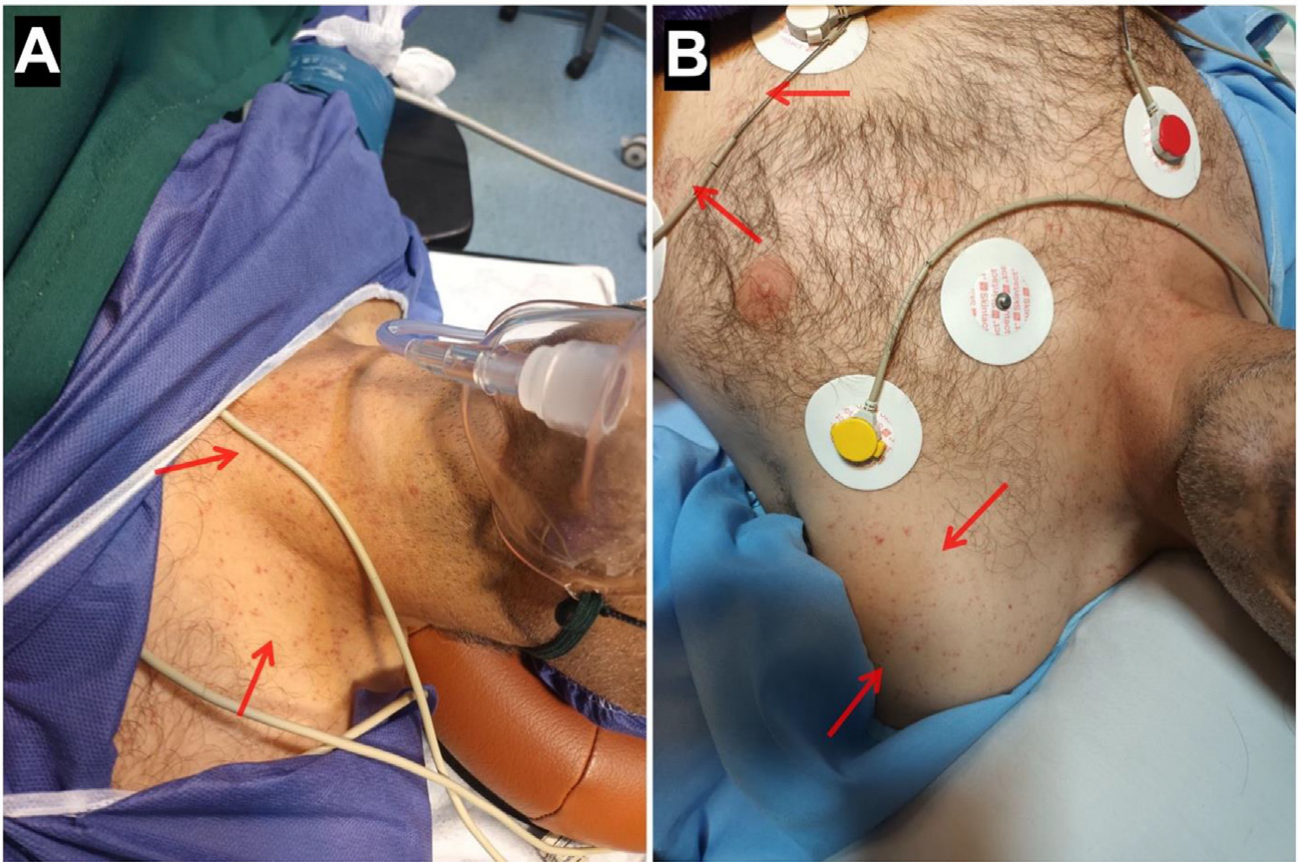
(A and B) Petechial lesions and purpura on the neck and chest (red arrows).

**Fig. 4:**
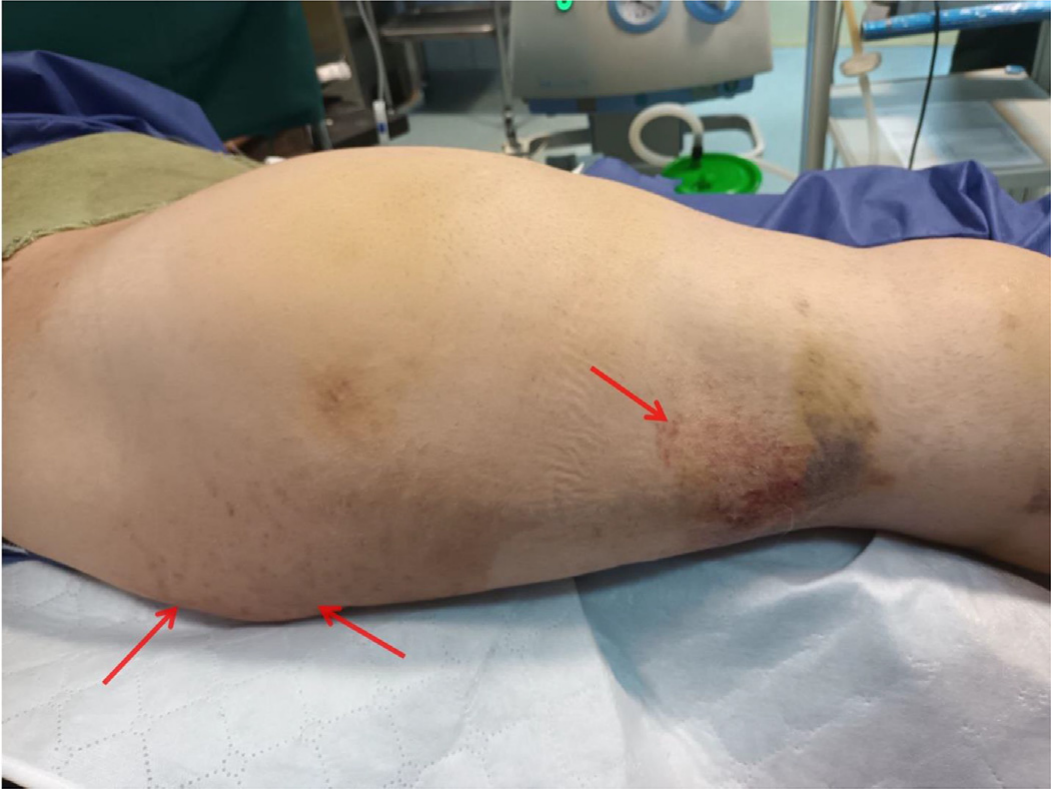
Petechial lesions and purpura on the right lower limb (red arrows).

Laboratory data showed a significant drop in hemoglobin (current Hb = 7.4 g/dL, postoperative Hb = 13.2 g/dL, reference range = 14-18 g/dL) and a rise in blood urea nitrogen (BUN) (current BUN = 63 mg/dL, postoperative BUN = 42 mg/dL, reference range = 15-45 mg/dL). Other lab data were within the normal range, and no leukocytosis or thrombocytopenia was observed. Oxygen therapy was initiated for the patient using a mask, and his oxygen saturation was within normal limits (SpO2 = 96%). The patient underwent an emergency CT angiography of the lungs, and enoxaparin at a dose of 60 mg every 12 hours subcutaneously was started for anticoagulation. Also, with a lower suspicion of fat embolism 150 mg of methylprednisolone was administered intravenously and an alternating pressure mattress was used to prevent deep vein thrombosis (DVT). The CT angiography was normal without any significant findings regarding pulmonary thromboembolism (PTE) (Fig. 2). The patient's blood was cross-matched and blood group analyzed, and 1 unit of packed red blood cells (PRBCs) was transfused due to the drop in hemoglobin. The patient became hemodynamically stable with normal vital signs (heart rate = 80, respiratory rate = 19, body temperature = 37.6°C). Twelve hours later, the patient developed petechial lesions and purpura on the neck, chest, and right lower limb (Fig. 3, Fig. 4). The suspicion for fat embolism was strengthened. All specific tests regarding Henoch-Schönlein Purpura (HSP) or IgA vasculitis and other types of vasculitis, including urinalysis, serum immunoglobulin A (IgA), coagulation tests, antineutrophil cytoplasmic antibodies (ANCA), serum complement levels (C3, C4), and liver function tests (LFTs), were insignificant and within normal ranges. So with the diagnosis of fat embolism, supportive care continued for the patient. After 24 hours, the patient became stable without the need for oxygen therapy, and his hemoglobin level was 8.4 g/dL (reference range = 14-18 g/dL).

## Patient outcome

After 2 days of monitoring, mobilization was started for the patient, and he was discharged from the hospital. All follow-up sessions since then have been good and showed no problems.

## Discussion

High-Risk patients for FES are typically younger males aged between 18 and 52, with studies showing a majority of patients are male with a mean age of around 36 years [9]. Smoking significantly increases susceptibility, as approximately 80.7% of FES patients are smokers [10]. The type and severity of femoral fractures are crucial, with high-energy fractures, especially type C in the AO classification, being more frequently associated with FES [9,11]. Additionally, patients sustaining multiple injuries or additional fractures, such as in the chest or skull, exhibit higher risk, evidenced by higher Revised Injury Severity Scores [7] in those with multiple traumas compared to isolated femoral fractures [9]. Surgical factors also play a role; delays in surgical intervention elevate FES risk, whereas early fixation of long bone fractures can be preventive [12]. Moreover, certain surgical techniques like intramedullary nailing are linked to a higher incidence of FES due to the potential for bone marrow manipulation leading to fat globules entering the bloodstream [11,12]. Finally, the severity of trauma, as indicated by higher RTSS, is positively correlated with the likelihood of developing fat emboli [9].

Clinical presentation and diagnosis of FES involve a complex interplay of symptoms, diagnostic criteria, imaging techniques, and laboratory tests. Patients typically present with a combination of respiratory distress, neurological symptoms, and a characteristic petechial rash, with symptoms usually appearing 1 to 3 days postinjury or surgery [[13], [14], [15]]. The Gurd and Wilson Criteria, which require meeting at least 1 major and 4 minor criteria, are widely used for diagnosis [15,16]. These criteria include respiratory symptoms (hypoxemia, dyspnea), neurological symptoms (confusion, focal deficits), petechial rash, and evidence of fat globules in the bloodstream or tissues. Differential diagnosis is crucial, necessitating the exclusion of other conditions with similar presentations [14,17]. In our case, the patient fulfilled 2 major criteria of the Gurd and Wilson Criteria for Fat Embolism Syndrome: Respiratory distress (tachypnea and ARDS) and Petechial rash, along with 3 minor criteria: Tachycardia (heart rate = 130), Fever (38.5°C), and an acute drop in hemoglobin (from 13.2 to 7.4 g/dL) (Table 1). Another widely used criteria is the Schonfeld Fat Embolism Index, which assigns points to symptoms for diagnosing fat embolism syndrome (FES): petechial rash (5 points), chest X-ray changes (4 points), hypoxia (3 points), and CNS symptoms, tachycardia, fever, retinal changes, urinary fat globules, anemia, and thrombocytopenia (1 point each). A score of 5 or more suggests FES [7]. In our case, according to the Schonfeld Fat Embolism Index, the patient met the following criteria: Petechial rash (5 points), Tachypnea (1 point), Tachycardia (1 point), and Fever (1 point). The total score is 8, indicating a high likelihood of fat embolism syndrome (FES) (Table 2).

**Table 1:** The Gurd and Wilson criteria for the diagnosis of fat embolism syndrome (FES).

Major criteria	Minor criteria
Respiratory distress	Tachycardia (>110 bpm)
Cerebral symptoms in nonhead injury patients	Fever (>38.5°C)
Petechial rash	Jaundice
	Renal changes
	Retinal changes
	Drop in hemoglobin
	New onset thrombocytopenia
	Elevated erythrocyte sedimentation rate
	Fat macroglobulinemia

A diagnosis of FES requires 1 major criterion and 4 minor criteria. In our case, the patient fulfilled 2 major criteria and 3 minor criteria.

**Table 2:** Schonfeld fat embolism index, which assigns a score based on clinical criteria for diagnosing fat embolism syndrome.

Criteria	Score
Petechiae	5
Chest X-ray changes (diffuse alveolar infiltrates)	4
Hypoxemia (PaO₂ <9.3 kPa)	3
Fever (>38°C)	1
Tachycardia (>120 bpm)	1
Tachypnea (>30 bpm)	1
Confusion	1

A score of 5 or more strongly suggests the presence of fat embolism syndrome. Our patient scored 8 points, indicative of a strong likelihood of FES.

Imaging techniques play a vital role in diagnosis, with pulmonary CT scans revealing characteristic findings such as ground-glass opacities and patchy consolidations [16]. In our patient, despite a high Schonfeld Fat Embolism Index score of 8, the chest X-ray and CT angiography of the lungs showed no positive findings for any condition, including FES. This discrepancy highlights the possibility of divergence between clinical scoring systems and imaging results in diagnosing fat embolism syndrome, underscoring the need for careful clinical judgment. Recent studies also suggest the potential utility of Point-of-Care Ultrasound (POCUS) in identifying right ventricular dysfunction associated with FES [18]. Laboratory tests, including blood tests for thrombocytopenia and anemia, as well as arterial blood gas analysis, further aid in diagnosis and assessment of disease severity [14,15]. Early recognition and comprehensive evaluation using these various diagnostic tools are essential for effective management of FES.

Effective management of FES involves a combination of preventive, diagnostic, and supportive strategies. Early stabilization of long bone fractures through surgical intervention is crucial, as studies have shown that timely surgery reduces the incidence of FES; damage control orthopedics may be employed in severely traumatized patients to prioritize initial stabilization over definitive repair [19]. Minimizing surgical trauma to reduce fat globule release into the bloodstream is also important. Early diagnosis relies on vigilant monitoring for symptoms such as respiratory distress, altered mental status, and petechial rash, which typically appear within 1-3 days postsurgery [20,21]. The Gurd and Wilson criteria aid in diagnosis, although a high index of suspicion is necessary, especially in high-risk patients. Supportive care for FES includes respiratory support, such as oxygen therapy or mechanical ventilation, to manage pulmonary complications [21,22], along with fluid management and vasopressors for hemodynamic instability [22]. The use of corticosteroids to reduce inflammation is a topic of ongoing debate, with some studies suggesting potential benefits but without universal acceptance as a standard treatment [19,20]. Finally, rehabilitation is a critical component of recovery, involving transfer to specialized facilities for continued care and physical therapy to restore limb function [22].

## Conclusion

Fat embolism syndrome (FES) is a rare but serious complication that can arise after long bone fractures or orthopedic surgeries. Early recognition and diagnosis are crucial to preventing severe complications. This case underscores the importance of clinical vigilance, as FES can be suspected based on clinical symptoms, even when imaging results are inconclusive. The Gurd and Wilson criteria and the Schonfeld Fat Embolism Index are useful diagnostic tools in such cases. Supportive care, including respiratory management and vigilant monitoring, plays a critical role in stabilizing patients and ensuring positive outcomes.

## Patient consent

Written informed consent was obtained from the patient for publication and any accompanying images. A copy of the written consent is available for review by the editor-in-chief of this journal on request.
